# Simultaneous spontaneous bilateral quadriceps tendon rupture with secondary hyperparathyroidism in a patient receiving hemodialysis: A case report

**DOI:** 10.1097/MD.0000000000014809

**Published:** 2019-03-08

**Authors:** Weiqian Wu, Chongyang Wang, Jianwei Ruan, Haibao Wang, Yang Huang, Wenbiao Zheng, Fanghu Chen

**Affiliations:** a Department of Orthopedics; b Department of Respiration, Taizhou Municipal Hospital, Taizhou, Zhejiang, China.

**Keywords:** hemodialysis, hyperparathyroidism, quadriceps tendon, rupture

## Abstract

**Rationale:**

Simultaneous spontaneous bilateral quadriceps tendon rupture is a rare orthopedic injury; its initial diagnosis is misdiagnosed in up to 50% of patients with secondary hyperparathyroidism. Early diagnosis and surgical repair are important to achieve an excellent functional outcome.

**Patient concerns:**

We report a case of simultaneous spontaneous bilateral quadriceps tendon rupture associated with secondary hyperparathyroidism.

**Diagnosis:**

Magnetic resonance imaging showed that the quadriceps tendon was completely ruptured at the osteotendinous junction. We then found bilateral quadriceps tendon rupture during the operation.

**Interventions:**

The patient underwent successful tendon repair surgery.

**Outcomes:**

The 31-year-old female patient regained full active movement of both knee joints and was able to participate in her activities of daily living.

**Lessons:**

Simultaneous spontaneous bilateral quadriceps tendon rupture in a patient with secondary hyperparathyroidism (undergoing hemodialysis) is a rare orthopedic injury that can be easily overlooked at the initial presentation. Early diagnosis and surgical repair is important to achieve an excellent functional outcome. For patients with secondary hyperparathyroidism receiving hemodialysis, strict systematic treatment of hyperparathyroidism is needed to prevent rupture or re-rupture of the quadriceps tendon.

## 1 Introduction

Spontaneous simultaneous bilateral quadriceps tendon rupture is a rare orthopedic injury in patients with secondary hyperparathyroidism who are receiving hemodialysis. The initial diagnosis of this injury is missed in up to 50% of cases. Moreover, it is complicated by concurrent use of steroids, quinolones, and statins^[1]^; comorbidities including chronic renal disease and hyperparathyroidism^[2]^; diabetes, rheumatoid arthritis, and obesity^[3]^; gout, systemic lupus erythematosus, and hypertension^[4]^; calkaptonuria^[5]^; polyneuropathy^[6]^; plus other unknown factors. Although the exact mechanism responsible for the rupture is controversial, it is primarily affected by secondary hyperparathyroidism associated with long-term hemodialysis treatment. Timely surgical repair is the recommended standard treatment, which leads to superior outcomes compared to delayed repair.^[7]^ Herein, we report a case of spontaneous concurrent bilateral rupture of quadriceps tendon in a patient with secondary hyperparathyroidism who was receiving hemodialysis.

## 2 Case report

Informed written consent was obtained from the patient for publication of this case report and accompanying images. Ethics committee approval was waived because this is a case report.

A 31-year-old female was admitted to the Department of Orthopedics in November, 2017, complaining of an inability to actively extend her bilateral knees. During detailed questioning, she stated that both her knees were “giving way” while she was walking downstairs since 6 months ago. Since then she was unable to bear weight without help and to actively extend both knees. She was sent to our emergency room for X-ray examinations; however, there was no obvious finding after viewing plain radiographs of both knees. She was diagnosed with ligament damage and both knees were immobilized to the extension position. She had no history of injury or long-term use of medications. Two weeks later, she presented to the Department of Orthopedics and again left without a clear diagnosis. The patient had received hemodialysis at our hospital over the past 5 years due to uremia.

**Figure 1:**
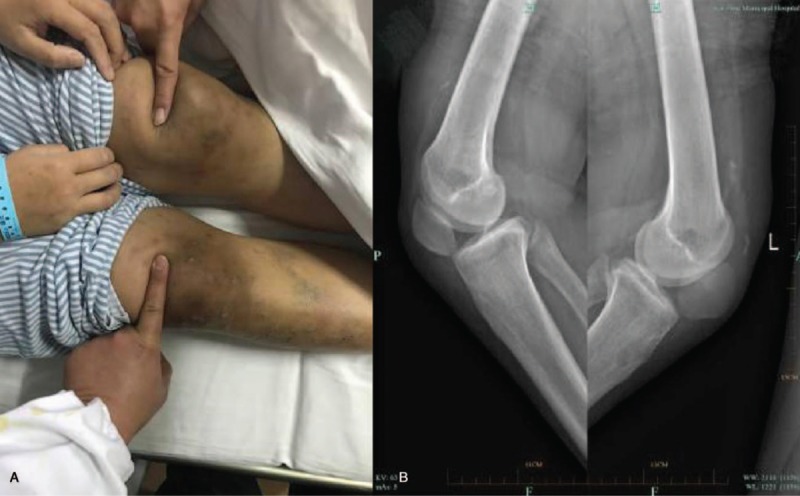
(A) Photograph of both knees revealing the bilateral soft tissue gap in the suprapatellar region. (B) Bilateral lateral radiography of her knees revealed inferior displacement of both patellas with irregular calcified deposits in the suprapatellar region (quadriceps tendon).

**Figure 2:**
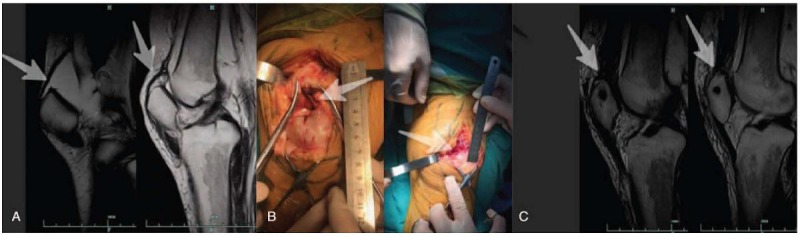
(A) Preoperative T2-weight sagittal MRIs of both knees clearly show the rupture at the osteotendinous junction as indicated by the arrows. (B) Intraoperatively, bilateral complete rupture between quadriceps tendon and patella was identified. (C) Postoperative sagittal T2-weighted MRIs of the knees revealed the normal continuity of the repaired quadriceps tendon with the superior pole of patella. MRIs = magnetic resonance images.

After admission to our department, a detailed physical examination showed a palpable gap above in both suprapatellar regions (Fig. 1A). Bilateral lateral radiography of her knees revealed inferior displacement of both patellas with irregular calcified deposits observed in the suprapatellar region (Fig. 1B). Magnetic resonance imaging (MRI) showed that the quadriceps tendon was completely ruptured at the osteotendinous junction (Fig. 2A). The serum parathyroid hormone (PTH) level was 1838.4 pg/ml (normal range 15–68.3 pg/ml), serum calcium 2.23 mmol/L (normal range 2–2.75 nmol/L), and serum phosphorus 2.1 mmol/L (normal range 0.87–1.45 mmol/L).

A diagnosis of bilateral quadriceps rupture and secondary hyperparathyroidism was made. A longitudinal incision was made overlying the patella and quadriceps tendon of each leg. Complete bilateral rupture between the quadriceps tendon and patella was identified at the osteotendinous junction (Fig. 2B).

We debrided the frayed soft tissue, refreshed the tendon ends, and roughened the proximal upper pole of the patella. Two vertical bone tunnels in the left patella and 3 vertical bone tunnels in the right patella were created. Two anchors (following drilling) were implanted into the left patella and 3 anchors into the right patella. Quadriceps tendons were sutured to the patella using the same technique used for Bunnel suture in full extension. The transverse diameter of the 4.5 mm tunnel was established on the upper third of the patella using the tunnel guide. We took the autologous semitendinosus and gracilis tendon and passed them through the transverse tunnel. Meanwhile, we established the tendon tunnel at the quadriceps tendon, inserted the transplanted tendon into the tunnel, and strained and sutured the ends of the transplanted tendon.

Reinforcement suturing was eventually carried out with a nonabsorbable suture between the transplanted tendon and surrounding tissue. Intraoperatively, both knees could be flexed up to 110° without causing tension to the sutured quadriceps tendon. Postoperative T2-weighted sagittal magnetic resonance images of bilateral knees revealed normal continuity of the repaired quadriceps tendon with the superior pole of the patella (Fig. 2C).

Long-leg cast immobilization was maintained for 6 months after surgery in extension. During this period, quadriceps isometric strengthening exercise, gentle patella mobilization, and ankle resistive exercise were performed. Meanwhile, we consulted the nephrologist and general surgeon. Considering the patient's age and side effects of parathyroidectomy, the general surgeon recommended that medical treatment be started first. As the serum phosphorus was higher than 1.8 mmol/L, oral calcium could produce a large amount of calcium phosphate resulting in soft tissue calcification. Therefore, the nephrologist recommended phosphorus reduction treatment. However, due to the high medical costs, the patient refused this additional treatment.

**Figure 3:**
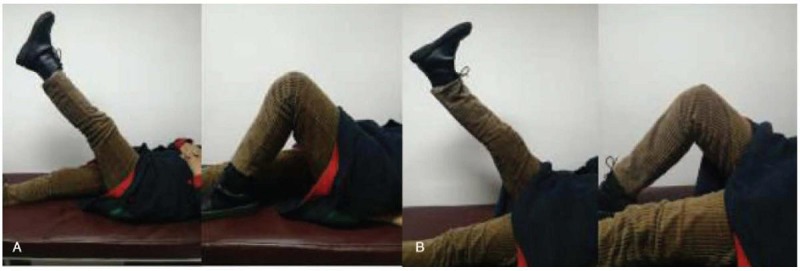
The patient regained full active movement of both knee joints and was able to participate in her activities of daily living.

After the casts were removed, progressively passive and active flexion of both knees was encouraged. At the 3-month follow up visit, joint activity of the knees increased to 0°/115° on the left and 0°/100° on the right. The strength of the bilateral quadriceps muscle was grade V (normal) on the Medical Research Council scale. The patient regained full active movement of both knee joints and was able to participate in her activities of daily living (Fig. 3). The duration of follow-up was 15 months and we monitored the PTH level every 3 months after the operation.

## 3 Discussion

The first case of bilateral simultaneous rupture of quadriceps tendon in a patient with secondary hyperparathyroidism undergoing chronic hemodialysis was reported by Preston and Adicoff in 1962.^[8]^ Since then, more cases of spontaneous simultaneous rupture of the bilateral quadriceps tendon in patients with chronic renal failure have been reported in the literature.^[2,8–15]^ This increased prevalence could be attributed to the more widespread use of hemodialysis, which has extended life expectancy and improved the quality of life in patients with chronic renal failure. However, it has also increases the incidence of secondary complications. Here we reported a case of spontaneous and simultaneous bilateral quadriceps tendon rupture in a patient with chronic renal failure undergoing hemodialysis.

The exact mechanism responsible for bilateral quadriceps tendon rupture remains controversial and has evolved from connective tissue elastosis to a multifactorial compound primarily affected by secondary hyperparathyroidism resulting from long-term hemodialysis treatment.^[16]^ Chronic acidosis, accumulation of uremic toxin, malnutrition, β_2_-amyloidosis, impaired collagen metabolism, and secondary hyperparathyroidism may also contribute to the development of spontaneous quadriceps tendon rupture in patients with chronic renal failure.

Decreased glomerular filtration rate leads to hypocalcemia and retention of phosphorus, which stimulates PTH release.^[17]^ High PTH stimulates osteoclast activation at subtendinous sites and contributes to secondary weakness at the osteotendinous junction as well as dystrophic calcification. Moreover, repeated minor fracture at the tendon insertion site can cause a final total rupture with spontaneous or minor trauma ^[13]^.

In our case, the patient suffered a sudden acute pain in the bilateral suprapatellar region and failed to actively extend her knees along with a palpable gap above the patella. This is highly suggestive of bilateral quadriceps tendon rupture. Unfortunately, the patient was not diagnosed in a timely fashion as 6 months had elapsed thus missing the best time to operate.

The diagnosis of bilateral quadriceps tendon rupture is mainly based on clinical manifestations and history. The typical clinical manifestations include sudden pain at the time of rupture above the patella, a palpable suprapatellar gap, inability to actively extend the knee, normal knee flexion, and passive range of motion. However, prompt precise diagnosis of complete quadriceps tendon rupture is often challenging for the following reasons:

(1) it is a rare orthopedics injury and the diagnosis of quadriceps tendon rupture may not be initially considered;
(2) owing to the presence of swelling and hemarthrosis, a palpable suprapatellar gap may not be identified and knee pain could be attributed to many different causes;
(3) no obvious trauma observed at the time of rupture; and
(4) when the rupture is bilateral, no obvious difference was observed in comparative physical examination.

To avoid a misdiagnosis of this rare disease, imaging techniques such as MRI and ultrasound can assist in confirming the diagnosis. MRI is the most preferred method to investigate tendon rupture despite its high cost and limited availability in less developed countries. MRI was used to confirm the diagnosis preoperatively and the good continuity of repaired tendon postoperative before the patient was discharged. Ultrasonography is a less expensive and important tool, which can be quickly and easily obtained, but is operator dependent.

Surgical repair is the recommended standard treatment for complete rupture of bilateral quadriceps tendon and early surgical repair will result in superior outcomes compared to delayed repair.^[7,10]^ We reattached the ruptured tendon onto the patella to recover the continuity of quadriceps tendon by using nonabsorbable sutures or an anchor and we used autologous semitendinosus and gracilis tendon to further reduce tension and enhance strength.

In a patient with secondary hyperparathyroidism receiving hemodialysis, strict treatment for hyperparathyroidism is needed to prevent further spontaneous tendon rupture. Total parathyroidectomy (with or without autotransplantation of part of the parathyroid gland in recalcitrant cases), early use of vitamin D analogs, phosphate binders, and calcimimetics are recommended to prevent future tendon rupture. For patients who refused treatment for hyperparathyroidism, we should pay attention to the occurrence of further spontaneous tendon rupture and infection. In our case report, the incision took almost 6 months to heal.

In summary, spontaneous simultaneous bilateral rupture of quadriceps tendon in a patient with secondary hyperparathyroidism undergoing hemodialysis is a rare orthopedic injury that can be easily overlooked at the initial presentation. This case report emphasized the importance of diagnosing quadriceps tendon rupture early. Early diagnosis and surgical repair are important to achieve an excellent functional outcome. For patients with secondary hyperparathyroidism receiving hemodialysis, strict systematic treat measures of hyperparathyroidism are needed to prevent rupture or re-rupture of the quadriceps tendon.

## Author contributions

**Data curation:** Jianwei Ruan, Yang Huang.

**Formal analysis:** Jianwei Ruan, Haibao Wang, Wenbiao Zheng.

**Funding acquisition:** Haibao Wang.

**Investigation:** Weiqian Wu, Chongyang Wang.

**Methodology:** Weiqian Wu, Chongyang Wang, Yang Huang, Fanghu Chen.

**Project administration:** Weiqian Wu, Wenbiao Zheng, Fanghu Chen.

**Supervision:** Chongyang Wang.
